# *Limosilactobacillus fermentum* MG4294 and *Lactiplantibacillus plantarum* MG5289 Ameliorates Nonalcoholic Fatty Liver Disease in High-Fat Diet-Induced Mice

**DOI:** 10.3390/nu15082005

**Published:** 2023-04-21

**Authors:** Ji Yeon Lee, Minju An, Huijin Heo, Jeong-Yong Park, Junsoo Lee, Chang-Ho Kang

**Affiliations:** 1 MEDIOGEN, Co., Ltd., Biovalley 1-ro, Jecheon-si 27159, Republic of Korea; ljy341@naver.com (J.Y.L.); jiy0900@daum.net (J.-Y.P.); 2 Department of Food Science and Biotechnology, Chungbuk National University, Cheongju-si 28644, Republic of Korea; juju7890@naver.com (M.A.); pltreasure11@gmail.com (H.H.)

**Keywords:** *Lactobacillus*, probiotics, NAFLD, HFD, hepatocytes

## Abstract

Non-alcoholic fatty liver disease (NAFLD) is the most common liver disease and the leading cause of liver-related deaths worldwide. It has been established that microorganisms are involved in the interaction between the intestinal lumen and the liver; therefore, studies on probiotics as potential candidates are increasing. This study evaluated the effects of *Limosilactobacillus fermentum* MG4294 and *Lactiplantibacillus plantarum* MG5289 on NAFLD. The MG4294 and MG5289 reduced lipid accumulation in FFA-induced HepG2 by suppressing the adipogenic proteins through the regulation of AMP-activated protein kinase (AMPK). The administration of these strains in the HFD-induced mice model lowered body weight, serum aspartate aminotransferase (AST), alanine aminotransferase (ALT), and cholesterol levels. In particular, MG4294 and MG5289 restored liver TG and TC to normal levels by lowering lipid and cholesterol-related proteins via the modulation of AMPK in the liver tissue. In addition, the administration of MG4294 and MG5289 reduced pro-inflammatory cytokines (tumor necrosis factor (TNF)-α and interleukin (IL)-1β-, and IL6) in the intestinal tissues of the HFD-induced mouse model. In conclusion, MG4294 and MG5289 can be presented as probiotics with the potential to prevent NAFLD.

## 1. Introduction

Non-alcoholic fatty liver disease (NAFLD) is a common liver disease worldwide and is closely related to metabolic syndromes. In addition, it was recently known as metabolic (dysfunction) associated fatty liver disease (MAFLD) [1,2]. NALFD is characterized by a total liver fat greater than 5% [3]. NAFLD occurs when free fatty acids (FFA) in the body rapidly increase, leading to increased free fatty acid absorption and triglyceride (TG) biosynthesis in the liver [4]. NAFLD can lead to hepatocellular damage, fibrosis, and even apoptosis due to the excessive accumulation of intracellular lipids and oxidative stress, inflammation, and mitochondrial dysfunction [5]. Despite its diverse symptomatic etiology, there are no Food and Drug Administration (FDA)-approved drugs to treat NAFLD [6]. Therefore, it is necessary to develop safe, functional foods that can prevent NAFLD.

Probiotics, defined as living microorganisms that benefit human health, are emerging as a new strategy for NAFLDs [7]. In patients with NAFLD, dysbiosis caused by alteration of the gut microbiota indicates liver diseases, such as hepatic steatosis and hepatitis, mainly through the gut–liver axis pathway [8]. Dysbiosis in NAFLD can accelerate NAFLD-related diseases by compromising the intestinal barrier and transporting pathogen-generated products and inflammatory cytokines to the liver via the hepatic portal vein, a pathway connecting the intestine and liver [9]. In clinical studies, it was reported that probiotic intake improved the symptoms of NAFLD by reversing intestinal dysbiosis [8]. Therefore, probiotics can be used as a preventive agent for NAFLD by improving the gut microbiome.

This study investigated the mechanism underlying the ameliorating effect of *Limosilactobacillus fermentum* MG4294 and *Lactiplantibacillus plantarum* MG5289 on FFA-induced HepG2 cells and mice with NAFLD induced by a high-fat diet. In addition, to confirm whether *L. fermentum* MG4294 and *L. plantarum* MG5289 improve NAFLD based on the gut –liver axis, pro-inflammatory cytokines in the intestinal tissue were measured, and the possibility of safe probiotics was proven.

## 2. Materials and Methods

### 2.1. Preparation of Sample and Strains Culture

Probiotics, *L. fermentum* MG4294 (human origin) and *L. plantarum* MG5289 (food origin), have been stored and provided by MEDIOGEN Co., Ltd. (Jechon, Republic of Korea). *L. fermentum* MG4294 and *L. plantarum* MG5289 were analyzed by 16S rRNA sequencing (SolGen, Co., Ltd., Daejeon, Republic of Korea) for identification, and cultured in De Man, Rogosa, and Sharpe (MRS) broth (Difco, Detroit, MN, USA). For the in vitro study, probiotic pellets were adjusted at 1 × 10^9^ CFU/mL in PBS and then homogenized using a homogenizer (KFS-150N, KORPROTECH Co., Ltd., Seoul, Republic of Korea). For the animal study, probiotic pellets were harvested by centrifugation, freeze-dried, and mixed with maltodextrin for adjustment at 5 × 10^10^ CFU/g [10].

### 2.2. Cell Culture

HepG2, human-derived hepatocytes (American Type Culture Collection, Rockville, MD, USA) were incubated in Dulbecco’s modified Eagle medium (DMEM, Gibco, Grand Island, NY, USA) containing 100 U/mL penicillin and 100 μg/mL streptomycin (PS, Gibco), and 10% heat-inactivated fetal bovine serum (FBS, Gibco) in 5% CO_2_-humidified air at 37 °C.

#### 2.2.1. FFA Mixture-Induction in HepG2 Cells

The FFA mixture was prepared by mixing palmitate and oleate (ratio of 1:2) in a sodium hydroxide (NaOH, 50 mM), respectively, and maintained at 70 ℃ for 30 min to prepare a suspension. The 10% bovine serum albumin (BSA) was mixed with tertiary distilled H_2_O and maintained at 55 °C. Thereafter, 10% BSA was mixed with the FFA solution and combined at 55 °C for 30 min to form 10 mM FFA/1% BSA solution [11].

#### 2.2.2. Cell Viability and Oil Red O Staining Assay

To evaluate cell viability, cells were dispensed in a 96-well plate (2 × 10^5^ cells/well), overnight. The FFA mixtures (500 μM) with *L. fermentum* MG4294 and *L. plantarum* MG5289 were treated for 24 h in each well. The 3-(4,5-Dimethylthiazol-2-yl)-2,5-diphenyl-tetrazolium bromide (MTT, 5 mg/mL) solution was added to each well and incubated for 2 h. Formazan crystals produced from viable cells were re-dissolved in dimethyl sulfoxide (DMSO). The absorbance at 550 nm was evaluated using a microplate reader (BioTek, Winooski, VT, USA).

For Oil Red O staining, HepG2 cells were cultured in a 12-well plate at 2 × 10^5^ cells/well for 24 h. The FFA mixture (500 μM) with cell extract of *L. fermentum* MG4294 and *L. plantarum* MG5289 in DMEM was treated in each well, overnight. Thereafter, the medium was removed, washed by phosphate-buffered saline (PBS), and cells were fixed using 10% formaldehyde at 25 °C. The fixed cells were washed with distilled water and stained with Oil Red O staining reagent for 15 min. The stained cells were washed with tertiary distilled water, and the stained lipids were eluted from the cells with isopropanol (200 μL). The absorbance (510 nm) was measured using a microplate reader (BioTek).

### 2.3. Animals

C57BL/6 male mice (5 weeks old) were used in this study (ORIENTBIO Inc., Gyeonggi-do, Republic of Korea). All mice were housed in an environment with a controlled temperature of 21 ± 2 °C and humidity of 50 ± 20% under a 12 h light/dark cycle. During the experimental period, diet and drinking water were fed by free intake. All animals were approved by the Institutional Animal Care and Use Committee of the NDIC (Gwangju, Gyeonggi-do, Republic of Korea, P222017).

#### 2.3.1. Experimental Design and Treatments

**Figure 1:**
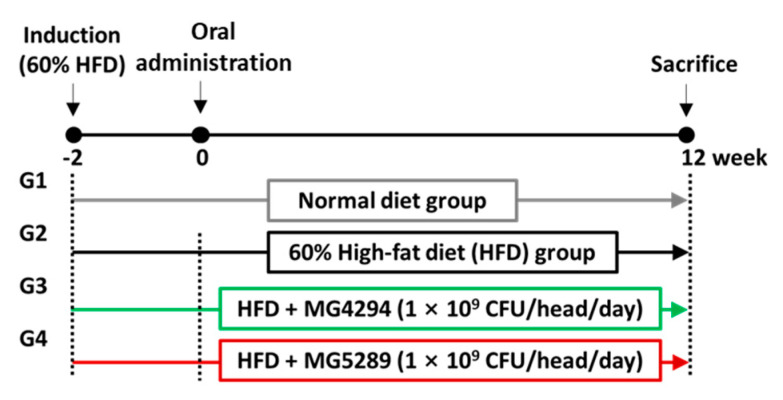
Animal experimental design for the effect of probiotics on NAFLD—induced mice (*n* = 6 per group).

The experimental schedule is shown in Figure 1. After 1 week of acclimatization, all animals (except for the six mice in the normal diet group) were fed a 60% HFD (D12492, Research Diets, Inc., New Brunswick, NJ, USA) 2 weeks early to induce obesity [12]. After the completion of obesity induction, their body weights were measured, and groups were divided so that the averages of the measured weight values between groups were similar. They were split into four groups (*n* = 6 per group) as follows: (1) normal (ND) group, (2) HFD group, (3) HFD + MG4294 (1 × 10^9^ CFU/head/day) group, and (4) HFD + MG5289 (1 × 10^9^ CFU/head/day) group. The mice were administered probiotics via oral gavage and were provided with 60% HFD feed for 12 weeks. Their body weights were checked once a week to observe the condition of the experimental animals. After 12 weeks, all animals were sacrificed by inhalational anesthesia with isoflurane, and their blood was drawn through the abdominal vena cava.

#### 2.3.2. Tissue, Plasma Collection, and Biochemical Parameter Analysis

The blood collected from the abdominal vena cava was divided into 0.6 mL SST microtainer tubes (BD, Franklin Lakes, NJ, USA), and the blood contained in the SST tubes was completely solidified. Each tube was centrifuged at 4 °C at 12,000 rpm for 2 min, and serum was collected. The liver was immediately harvested, rinsed with physiological saline solution, weighted, and stored at −70 °C until analysis. The triglyceride (TG), total cholesterol (TC), HDL—cholesterol (HDL—C), LDL—cholesterol (LDL—C), aspartate aminotransferase (AST), alanine aminotransferase (ALT), and glucose of the serum were analyzed by blood chemistry analyzer (AU480, Beckman Coulter, Germany). Hepatic TG (STA396, Cell Biolabs, San Diego, CA, USA) and TC (ab65390, Abcam, UK) were evaluated by commercial kits. The absorbance was measured at 450 nm (SpectraMax M2, Molecular Devices, San Jose, CA, USA).

#### 2.3.3. Histological Analysis

The liver tissue was fixed with 10% neutral buffered formalin and embedded in paraffin. Prepared paraffin blocks were cut into sections using a microtome (HM340E, Thermo-Scientific, Waltham, MA, USA) to a thickness of 4 μM. After removing the paraffin with xylene and 100–70% ethanol, the sections were stained with hematoxylin and eosin (H&E, BBC biochemical, Mount Vernon, WA, USA). Sections were examined under a light microscope (ECLIPSE 50i, Nikon, Tokyo, Japan) and photographed at 50 × magnification. The non-alcoholic fatty liver disease activity score (NAS) was performed using the previous report [13].

#### 2.3.4. Analysis of Pro-Inflammatory Cytokines

The colon tissue in Tissue Extraction Reagent I (Invitrogen, Waltham, MS, USA) was homogenized using BeadBug™ 6 (Benchmark Scientific, Sayreville, NJ, USA). The homogenates were centrifuged (10,000× *g* for 10 min at 4 °C), and a supernatant was used. Tumor necrosis factor (TNF)-α and interleukin (IL)-1β, IL-6 in colon tissue were analyzed by enzyme-linked immunosorbent assay kits (R&D systems, Minneapolis, MN, USA). The absorbance was measured at 450 nm (BioTek).

### 2.4. Protein Extraction

HepG2 cells were lysed by Pro-Prep™ sample buffer (iNtRON Biotechnology, Seongnam, Republic of Korea). The liver tissues were homogenized and prepared in Tissue Extraction Reagent I (Invitrogen) containing phosphatase and protease inhibitors (Gendepot, Katy, TX, USA). Cell and tissue lysates containing equal amounts of total proteins were determined using the Take3™ Multi-Volume Plate (Biotek) and Bradford assay (Gendepot).

### 2.5. Western Blotting

Western blot was performed as previously reported [14]. The proteins were separated by sodium dodecyl sulfate-polyacrylamide gel electrophoresis (SDS-PAGE) and transferred to a polyvinylidene fluoride (PVDF) membrane (Gendepot). The membrane was incubated following primary antibodies: p-AMP-activated protein kinase (AMPK), AMPK (Cell signaling, Beverly, MA, USA), sterol regulatory-element binding proteins (SREBP)1c (Santa Cruz Biotechnology, Dallas, TX, USA), fatty acid synthase (FAS; Cell signaling), peroxisome proliferator-activated receptor-γ (PPARγ; Cell signaling), CCAAT/enhancer-binding protein-α (C/EBPα; Cell signaling), SREBP1 (Abcam), SREBP2 (ABclonal, Wuhan, Hubei, China), cytochrome P450 family 7 subfamily a member 1 (CYP7A1; ABclonal), 3-hydroxy-3-methyl-glutaryl coenzyme A reductase (HMCGR; Santa Cruz Biotechnology), β-actin (Santa Cruz Biotechnology), and glyceraldehyde 3-phosphate dehydrogenase (GAPDH; Santa Cruz Biotechnology). The secondary antibodies, including Goat anti-Mouse IgG (H+L) and Goat anti-Rabbit IgG(H+L)-HRP, were obtained from Gen-depot. The blots were visualized using the West-Q Femto Clean enhanced chemiluminescence (ECL) solution (Gendepot) and LuminoGraph III Lite (ATTO, Tokyo, Japan), following the manufacturer’s instructions. Quantitative analysis was measured with CS Analyzer 4 (ATTO).

### 2.6. Safety Test as Probiotics

#### 2.6.1. Antibiotic Susceptibility Test

Antibiotic susceptibility test to determine the minimum inhibitory concentrations (MIC) of *L. fermentum* MG4294 and *L. plantarum* MG5289 was measured using ETEST (BioMérieux, Marcy-l’Étoile, France), which is mainly used for gradient strip tests [15]. ETEST was performed according to the manufacturer’s instructions. The strains were cultured in MRS broth and centrifuged at 4000 rpm. The pellets were diluted from 0.5 to 1.0 by McFarland buffer. Thereafter, inoculated Brain Heart Infusion agar (BHI, Difco) where strips were positioned and cultured for 48 h to interpret MIC.

#### 2.6.2. Hemolysis and Bile Salt Hydrolase (BSH) Activity

The *L. fermentum* MG4294 and *L. plantarum* MG5289 were plated on tryptic soy agar (Oxoid Ltd., Hampshire, UK) containing 5% sheep blood (MB cell, Seoul, Republic of Korea) and cultured at 37 °C. After 48 h, hemolysis was reported according to the color of the ring around the bacterial colony [16].

*L. fermentum* MG4294 and *L. plantarum* MG5289 were inoculated on MRS agar containing 0.5% (*w*/*v*) sodium glycodeoxycholate and 0.5% (*w*/*v*) taurodeoxycholate and incubate for 48 h, at 37 °C. BSH activity was judged by the formation of precipitates around the colonies. [17]

### 2.7. Statistical Analysis

All data were indicated by the mean ± standard error of the mean (SEM). The significant difference was performed through a one-way analysis of variance (ANOVA) followed by Fisher’s Least Significant Difference (LSD) test with *p* < 0.05 using the Statistical Package for the Social Sciences (SPSS) software (IBM, Armonk, NY, USA).

## 3. Results

### 3.1. Inhibitory Effect of L. fermentum MG4294 and L. plantarum MG5289 on Lipid Accumulation in FFA-Induced HepG2 Cells

**Figure 2:**
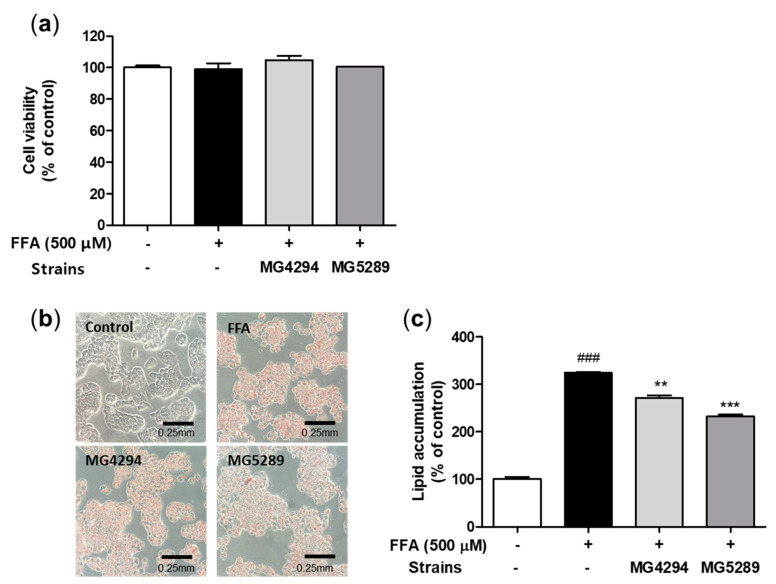
Cell viability and lipid accumulation of *L. fermentum* MG4294 and *L. plantarum* MG5289 in FFA-induced HepG2 cells. The cell viability (**a**), representative images of Oil Red O stain (**b**), and lipid accumulation (**c**) on FFA-induced HepG2 cells treated with MG4294 and MG5289. The data are expressed as mean ± SEM (*n* = 3). ^###^ *p* < 0.001 compared with control; ** *p* < 0.01 and *** *p* < 0.001 compared with FFA-induced control.

As a result of the cell viability of *L. fermentum* MG4294 and *L. plantarum* MG5289 treated with FFA in HepG2 cells, there was no significant change (Figure 2a). To confirm the lipid accumulation inhibitory effect of *L. fermentum* MG4294 and *L. plantarum* MG5289, Oil Red O staining was performed, and lipid droplets were observed through a microscope, as shown in Figure 2b,c. The contents of stained lipid droplets in FFA-induced HepG2 cells were more than that of the control, and the lipid accumulation was also significantly increased in FFA-induced HepG2 cells. In contrast, stained lipid droplets and accumulation were significantly decreased by treatment of *L. fermentum* MG4294 and *L. plantarum* MG5289 with FFA in HepG2 cells. 

### 3.2. Modulating of L. fermentum MG4294 and L. plantarum MG5289 on Lipid Metabolism-Related Factors in FFA-Induced HepG2 Cells

**Figure 3:**
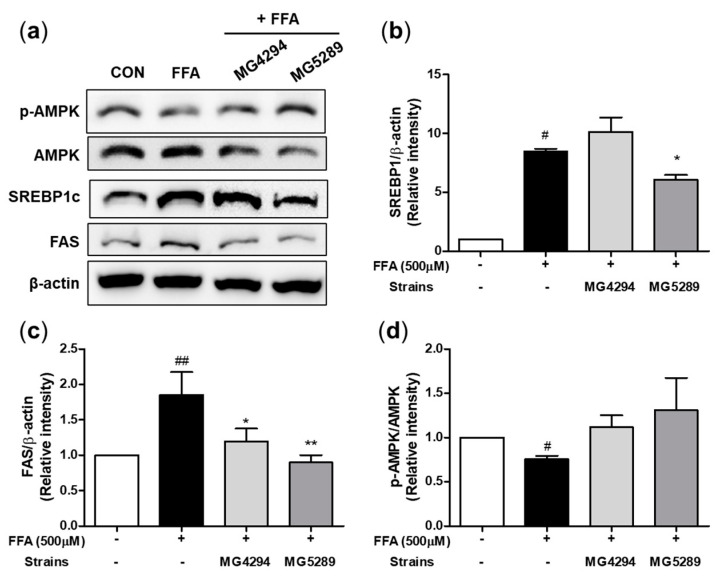
Effects of *L. fermentum* MG4294 and *L. plantarum* MG5289 on lipid metabolism in FFA-induced HepG2 cells. The representative blotting images (**a**) and protein expression levels of SREBP1c (**b**), FAS (**c**), and p-AMPK/AMPK (**d**) were analyzed via Western blotting. The β-actin and AMPK were used as loading control. The data are expressed as mean ± SEM (*n* = 3). ^#^ *p* < 0.05 and ^##^ *p* < 0.01 compared with control; * *p* < 0.05 and ** *p* < 0.01 compared with FFA-induced control.

To confirm the effect of *L. fermentum* MG4294 and *L. plantarum* MG5289 on FFA-induced HepG2 cells, the expressions of adipogenesis-related proteins were measured (Figure 3). Expression of SREBP1c (*p* < 0.05) and FAS (*p* < 0.01) was increased, and p-AMPK (*p* < 0.05) was significantly reduced by the FFA induction in HepG2 cells. The *L. plantarum* MG5289 significantly decreased the expression of SREPB1c (*p* < 0.05) and FAS (*p* < 0.01) and increased the p-AMPK; however, *L. fermentum* MG4294 significantly reduced only the expression of FAS (*p* < 0.05).

### 3.3. Effects of MG4294 and MG5289 on Weight Gain, Body, and Tissue Weight in HFD-Induced Mice

**Figure 4:**
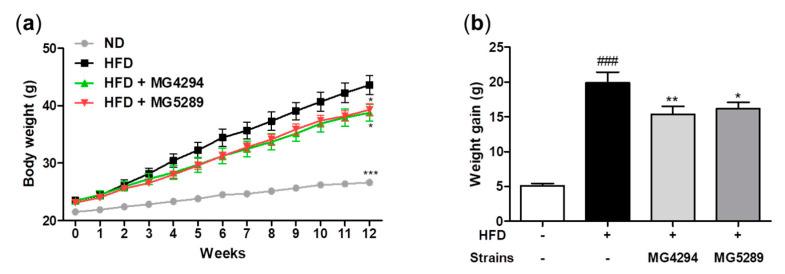
Administration of *L. fermentum* MG4294 and *L. plantarum* MG5289 alleviate body weight (**a**) and weight gain (**b**) in HFD mice. The data are expressed as mean ± SEM (*n* = 6). ^###^ *p* < 0.001 compared with ND group; * *p* < 0.05, ** *p* < 0.01 and *** *p* < 0.001 compared with HFD-group.

During the entire animal study period, no deaths or abnormal symptoms were observed due to the administration of probiotics. The body weight of *L. fermentum* MG4294 and *L. plantarum* MG5289 treated groups was less than that of the HFD group, respectively (Figure 4a). In addition, *L. fermentum* MG4294 and *L. plantarum* MG5289 treated groups showed a significant decrease in weight gain by 22.93% (*p* < 0.01) and 18.91% (*p* < 0.05) compared to the HFD group (Figure 4b).

### 3.4. Effects of MG4294 and MG5289 on Biochemical Parameters in Serum in HFD-Induced Mice

**Figure 5:**
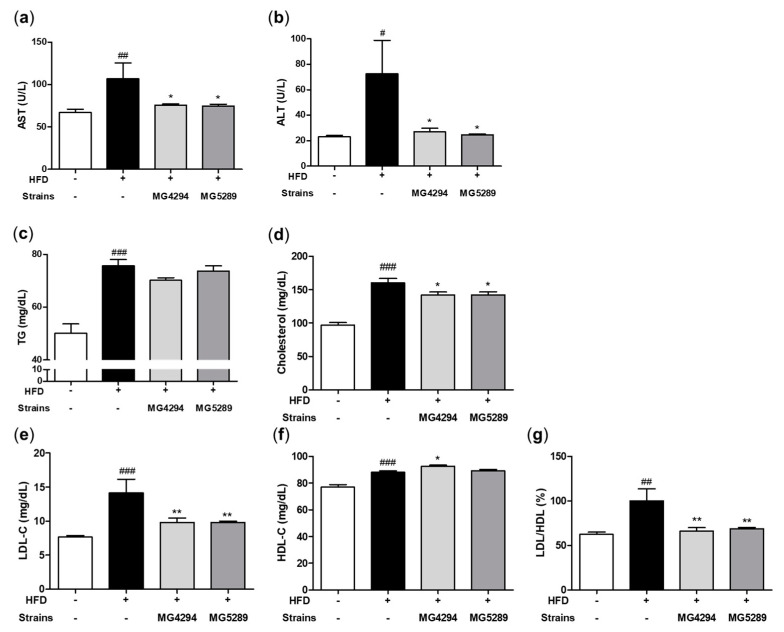
*L. fermentum* MG4294 and *L. plantarum* MG5289 improved AST (**a**), ALT (**b**), TG (**c**), TC (**d**), LDL-C (**e**), HDL-C (**f**), and LDL/HDL (**g**) in serum of HFD-induced mice. The data are expressed as mean ± SEM (*n* = 6). ^#^ *p* < 0.05, ^##^ *p* < 0.01 and ^###^ *p* < 0.001 compared with ND group; * *p* < 0.05 and ** *p* < 0.01 compared with HFD-group.

Serum analysis results for all biochemical parameters are shown in Figure 4. The levels of AST and ALT, indicators of liver toxicity enzyme, significantly decreased in the *L. fermentum* MG4294 and *L. plantarum* MG5289 treated groups compared to the HFD group (*p* < 0.05; Figure 5a,b).

The lipid indicators, TG, TC, and LDL-C were all significantly increased in the HFD group compared to the ND group (*p* < 0.001). In serum TG levels, *L. fermentum* MG4294 and *L. plantarum* MG5289 treated groups slightly decreased by 7.27% and 2.64%, compared to the HFD group (Figure 5c). Serum TC levels were decreased by the administration of *L. fermentum* MG4294 and *L. plantarum* MG5289, respectively (*p* < 0.05; Figure 5d). The *L. fermentum* MG4294 and *L. plantarum* MG5289 treated group significantly reduced LDL-C (*p* < 0.01) but had no effect on HDL-C levels (Figure 5e,f). The ratio of LDL-C and HDL-C accounted for a large portion of the TC reduction. When converted into a calculated ratio, there was a significant difference between *L. fermentum* MG4294 and *L. plantarum* MG5289 treated groups and the HFD group (*p* < 0.01; Figure 5g).

### 3.5. Effects of MG4294 and MG5289 on Liver Steatosis in HFD-Induced Mice

**Figure 6:**
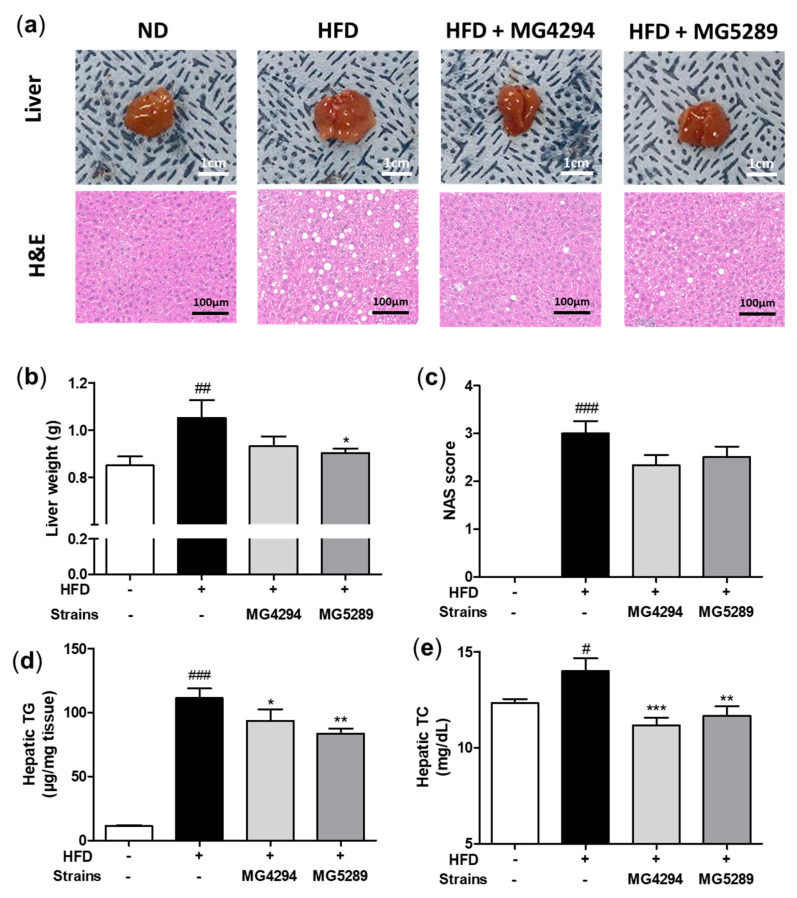
Treatment of *L. fermentum* MG4294 and *L. plantarum* MG5289 suppresses hepatic dysfunctions in HFD-induced mice. Representative images and microscopic H&E staining of the liver from different mice groups fed with HFD are shown (**a**). The liver weight (**b**) and NAS (**c**) were measured and calculated. Hepatic TG (**d**) and TC (**e**) were analyzed by ELISA kits. The data are expressed as mean ± SEM (*n* = 6). ^#^ *p* < 0.05, ^##^ *p* < 0.01, and ^###^ *p* < 0.001 compared with ND group; * *p* < 0.05, ** *p* < 0.01 and *** *p* < 0.001 compared with HFD-group.

To confirm the effect on the liver size, the livers of each group were observed (Figure 6a). The liver size of the *L. fermentum* MG4294 and *L. plantarum* MG5289 treated groups slightly decreased compared to the HFD group. H&E staining was performed to confirm the degree of lipid droplets in the liver tissue. The number and size of lipid droplets increased in the HFD group; however, when *L. fermentum* MG4294 and *L. plantarum* MG5289 were administered, that of lipid droplets decreased in liver tissue (Figure 6a).

The liver weight of the *L. fermentum* MG4294 and *L. plantarum* MG5289 treated groups decreased by 11.46% and 14.14% (*p* < 0.05) compared to the HFD group (Figure 6b). As a result of NAS, hepatic TG, and hepatic TC, which are indicators of liver dysfunction, a statistically significant increase was observed in the HFD group compared to the ND group (*p* < 0.001, *p* < 0.001, and *p* < 0.05; Figure 6b). All factors were decreased in the *L. fermentum* MG4294 and *L. plantarum* MG5289 treated groups, particularly in hepatic TG (*p* < 0.05) and hepatic TC (*p* < 0.001 and *p* < 0.01).

### 3.6. Effects of MG4294 and MG5289 on Lipogenic Markers in the Liver of HFD-Induced Mice

**Figure 7:**
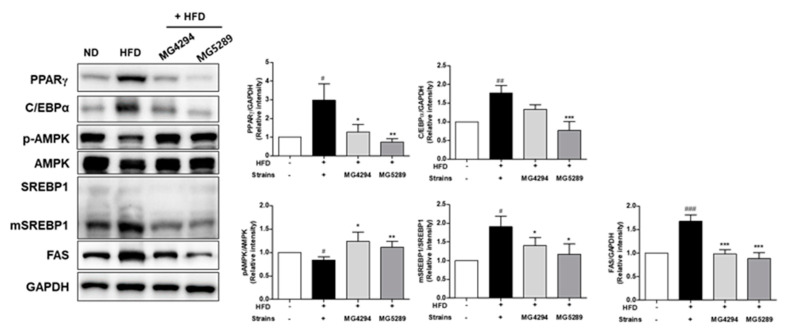
*L. fermentum* MG4294 and *L. plantarum* MG5289 modulate lipid metabolism-related factors in liver tissue of HFD-induced mice. The expression of p-AMPK was normalized by AMPK; all proteins except this one were normalized to GAPDH. The data are expressed as mean ± SEM (*n* = 6). ^#^ *p* < 0.05, ^##^ *p* < 0.01, and ^###^ *p* < 0.001 compared with ND group; * *p* < 0.05, ** *p* < 0.01, and *** *p* < 0.001 compared with HFD-group.

This study investigated whether the administration of *L. fermentum* MG4294 and *L. plantarum* MG5289 is involved in the expression of proteins-related lipogenesis and cholesterol regulation in liver tissue in HFD-induced mice (Figure 7). The expressions of proteins-related lipogenesis, such as PPARγ, C/EBPα, p-AMPK, mature (m)-SREBP1, and FAS, were significantly increased by HFD in the liver tissue of mice. However, when *L. fermentum* MG4294 and *L. plantarum* MG5289 were administered, that of the expressions were decreased.

### 3.7. L. fermentum MG4294 and L. plantarum MG5289 Affected Pro-Inflammatory Cytokine in the Intestinal Tissue of HFD-Induced Mice

**Figure 8:**
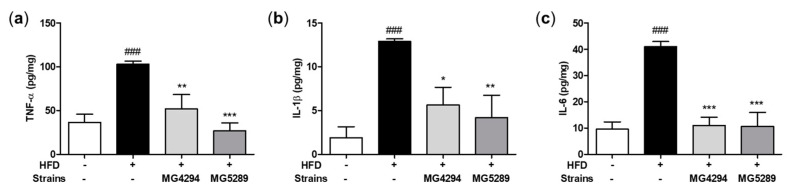
Effects of MG4294 and MG5289 on pro-inflammatory cytokines in HFD-induced mice. The TNF-α (**a**), IL-1β (**b**), and IL-6 (**c**) were analyzed by ELISA kits. The data are expressed as mean ± SEM (*n* = 6). ^###^ *p* < 0.001 compared with ND group; * *p* < 0.05, ** *p* < 0.01 and *** *p* < 0.001 compared with HFD-group.

The effect of treatments of *L. fermentum* MG4294 and *L. plantarum* MG5289 on pro-inflammatory cytokine levels in the intestinal tissues of mice induced by HFD was confirmed through ELISA analysis (Figure 8). In the HFD group, the levels of all cytokines, including TNF-α, IL-1β, and IL-6, were significantly increased in the intestinal tissues of the mice (*p* < 0.001). In contrast, *L. fermentum* MG4294 and *L. plantarum* MG5289 treated groups had significantly decreased levels of TNF-α (*p* < 0.01 and *p* < 0.001), IL-1β (*p* < 0.05 and *p* < 0.01), and IL-6 (*p* < 0.001) in intestinal tissues of HFD-induced mice.

### 3.8. Safety Test of L. fermentum MG4294 and L. plantarum MG5289

As a result of antibiotic resistance confirmation of the selected strains, both strains had an antibiotic resistance lower than the antibiotic resistance limit of the European Food and Drug Administration (EFSA), confirming that they are safe strains that do not have antibiotic resistance (Table 1).

As a result of confirming the hemolytic activity of *L. fermentum* MG4294 and *L. plantarum* MG5289, both strains have no hemolytic activity (γ-hemolysis) and no BSH precipitation, proving that they are safe for the host when ingested (Figure S1).

## 4. Discussion

NAFLD is the most common liver disease, with a very high prevalence estimated to affect more than 25% of the world population, including 1/3 of the population in the United States and Asia [19,20]. NAFLD is known to cause various liver diseases, including doubling the progression of liver cancer; however, it is still often overlooked [21]. Despite its significance, no synthetic drugs directly target NAFLD, and some drugs affect NAFLD indirectly; however, they have side effects such as an increased serum cholesterol, itching, and diarrhea [22]. In addition, significant uncertainty in the treatment period and prevention of NAFLD, and the most effective treatment methods, such as gastric bypasses, are very costly, causing major social and economic problems [23,24]. Probiotics can be used as a new strategy as Generally Recognized as Safe (GRAS) which is not synthetic drugs [25]. In general, the administration of probiotics effectively corrects dysbiosis in the gut microbiome [26]. Recently, the evidence for the association between dysbiosis in the gut and NAFLD has increased, and research on the ‘gut-liver axis’ has attracted attention [27]. Because 70–75% of the blood supply to the liver is supplied from the intestine through the portal vein, various metabolites and toxins produced in the intestine can affect liver health [28]. In particular, intestinal microorganisms can control the occurrence of NAFLD by lowering absorption using carbohydrates [29]. Notably, it has been reported that a HFD-fed mouse model forms a microbiome similar to that of mice with NAFLD, confirming the effect on the gut–liver axis in NAFLD [8]. In an HFD-induced mouse model, *L. plantarum* LC27, *Bi. longum* LC67, and *Lc. lactis* inhibit NAFLD by regulating the intestinal microflora, and *L. rhamnosus* GG and *L. paracasei* N1115 showed efficacy against NAFLD by regulating intestinal inflammatory cytokines and tight junctions [30,31,32,33]. In addition, it has been reported that the administration of *L. acidophilus*, *L fermentum*, *L. paracasei*, and *L. plantarum* significantly improved the disease state in patients with NAFLD [34]. In addition, HepG2 cell is an established in vitro model system to confirm NAFLD [35]. Therefore, this study investigated the improving effect *L. fermentum* MG4294 and *L. plantarum* MG5289 have on NAFLD on the FFA-induced HepG2 cells and HFD-induced mice model.

The HFD-induced mouse can be used as a preclinical model mimicking the metabolic and histological features of human NAFLD [24]. Intake of HFD for 12 weeks increases the body weight of mice, liver damage indexes such as AST and ALT, and TC in serum. [36]. Notably, the LDL/HDL ratio is an important indicator of atherosclerosis, which promotes the progression of liver fibrosis [24,37]. Administration of *L. fermentum* MG4294 and *L. plantarum* MG5289 markedly improved weight gain and lowered AST, ALT, and LDL/HDL in serum, demonstrating the lipid improvement effect in serum. In addition, the consumption of HFD exhibits hepatic steatosis, a typical histopathological feature of NAFLD [24]. As a result of H&E staining confirming the efficacy *L. fermentum* MG4294 and *L. plantarum* MG5289 on the degree of adipogenesis in liver tissue, it was visually observed that lipid droplets were reduced. These results were confirmed to be consistent with the results of suppressing the lipid accumulation of *L. fermentum* MG4294 and *L. plantarum* MG5289 in HepG2 cells in which the formation of lipid droplets was induced by FFA. In addition, hepatic TG, and TC in mice, which act as major indicators for NAFLD, are increased by HFD [36]. As a result, when *L. fermentum* MG4294 and *L. plantarum* MG5289 were fed to mice with NAFLD induced by HFD, significant decreases in hepatic TG and TC were observed.

The major proteins involved in adipogenesis in the liver are PPARγ and C/EBPα [38]. Overexpression of PPARγ and C/EBPa induces lipid accumulation, an initiating step in NAFLD pathogenesis [38,39]. Thus, it is important to identify the regulation of the two proteins applicable to developing preventive and therapeutic agents for NAFLD [38]. In this study, the expression of PPARγ and C/EBPα, which were increased by HFD induction in the liver of mice, was improved by *L. fermentum* MG4294 and *L. plantarum* MG5289. SREBP1 is involved in lipid homeostasis in the liver and increases the protein expression of FAS [40]. Overexpressed FAS contributes to the pathogenesis of NAFLD by accumulating intrahepatic TG [41]. Administration of *L. fermentum* MG4294 and *L. plantarum* MG5289 significantly decreased the expression of SREBP1 and FAS in the liver of HFD-induced mice. Particularly, *L. plantarum* MG5289 reduced the expression of SREBP1 in FFA-induced HepG2 cells. In the liver, AMPK is activated by phosphorylation, which plays the role of a metabolic key in the regulation of both adipogenesis and lipogenesis [4]. As a result of confirming the expression of phosphorylated AMPK, *L. fermentum* MG4294 and *L. plantarum* MG5289 reversed the level lowered by HFD. The increased expression of p-AMPK by *L. fermentum* MG4294 and *L. plantarum* MG5289 was similarly confirmed in FFA-induced HepG2 cells. Therefore, it is found that administration of *L. fermentum* MG4294 and *L. plantarum* MG5289 reduced the expression of SREBP1 and FAS related to TG formation and suppressed the expression of PPARγ and C/EBPα, which are adipogenesis-related proteins, through phosphorylation of AMPK in the liver of HFD-induced mice, thereby affecting NAFLD. In addition, as previously reported, HFD induces dysbiosis and increased intestinal epithelial permeability, leading to intestinal inflammatory cytokines, which enter the liver through the hepatic portal vein and cause liver damage [27,28]. Intestinal inflammatory cytokines such as TNF-α, IL-1β, and IL-6, which contribute to the development and progression of NAFLD, were confirmed and improved by administration of *L. fermentum* MG4294 and *L. plantarum* MG5289. These results suggest that *L. fermentum* MG4294 and *L. plantarum* MG5289 can alleviate NAFLD by regulating the gut–liver axis.

Probiotics have traditionally been used as food and recognized as safe [25]. However, to commercially use the new strain, their safety must be established [42]. In general, methods for verifying the safety of strains include antibiotic resistance, hemolysis, and BSH precipitation confirmation [17]. When probiotics strains are killed by various antibiotics, the functionality of probiotics in humans is lowered [43]. In this respect, antibiotic resistance is recognized as a very important factor [43,44]. In addition, the antibiotic resistance of strains varies from strain to strain [44]. Therefore, as a result of confirming whether *L. fermentum* MG4294 and *L. plantarum* MG5289 were resistant to 16 commonly used antibiotics, it was confirmed that they met all MICs according to the EFSA guidelines. Because some pathogens cause hemolysis and lysis of red blood cells in humans, avoiding hemolysis is crucial for safety [17]. As a result of evaluating the hemolytic properties, both *L. fermentum* MG4294 and *L. plantarum* MG5289 had no activity, confirming safety. Precipitation of BSH is caused by hydrolase activity, which can be potentially harmful to the human host by causing DNA damage, promoting colon cancer, and forming gallstones [17]. *L. fermentum* MG4294 and *L. plantarum* MG5289 did not form a white ring, confirming that they were inactive.

In summary, we confirmed that *L. fermentum* MG4294 and *L. plantarum* MG5289 improve liver steatosis via AMPK signaling pathway in cell and animal models. However, it is unclear whether it has the same efficacy in humans. Therefore, further studies, such as clinical trials, are needed.

## 5. Conclusions

**Figure 9:**
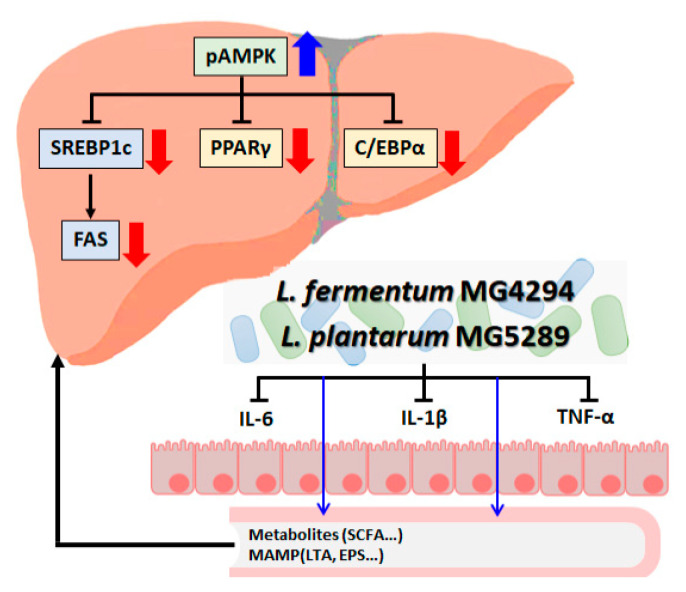
Effects of *L. fermentum* MG4294 and *L. plantarum* MG5289 on NAFLD by modulating of gut–liver axis. Thick blue arrows, increased expression by MG4294 and MG5289; thick red arrows, decreased expression by MG4294 and MG5289; thin blue arrow, generating metabolites by MG4294 and MG5289.

This study demonstrated the effect of *L. fermentum* MG4294 and *L. plantarum* MG5289 on NAFLD in vitro and in vivo. *L. fermentum* MG4294 and *L. plantarum* lowered the possibility of NAFLD exacerbation by suppressing intestinal inflammatory cytokines and activated AMPK phosphorylation in liver tissue to regulate the expression of proteins involved in lipid and cholesterol synthesis (Figure 9). In addition, to evaluate the safety of probiotics, antimicrobial, hemolysis, and BSH activities were performed to prove that *L. fermentum* MG4294 and *L. plantarum* MG5289 were safe. Based on the results of this study, *L. fermentum* MG4294 and *L. plantarum* MG5289 are safe probiotics and can be used as functional food or pharmaceutical materials to improve NAFLD.

## Figures and Tables

**Table 1:** Minimum inhibitory concentrations (MIC) of antibiotics of *L. fermentum MG4294* and *L. plantarum MG5289,* and cut-off value of the European Food Safety Authority (EFSA).

Antibiotics (μg/mL)	*L. fermentum* MG4294		*L. plantarum* MG5289	
	MIC (μg/mL)	EFSA Cut-Off Value *	MIC (μg/mL)	EFSA Cut-Off Value
Ampicillin	0.094	2	1	2
Gentamycin	0.19	16	0.38	16
Kanamycin	4	64	8	64
Streptomycin	6	64	12	64
Tetracycline	1.5	8	12	32
Chloramphenicol	3	4	6	8
Erythromycin	0.25	1	0.125	1
Clindamycin	0.016	4	0.19	2

* The cut-off value was listed according to EFSA guidelines [18].

## Data Availability

The authors declare that all data and materials support published claims and comply with field standards.

## Supplementary Materials

The following supporting information can be downloaded at: https://www.mdpi.com/article/10.3390/nu15082005/s1, Figure S1: Hemolysis (**a**) and BSH activities (**b**) of *L. fermentum* MG4294 and *L. plantarum* MG5289.
