# Supplementary figures and images for: Limosilactobacillus fermentum MG4294 and Lactiplantibacillus plantarum MG5289 Ameliorates Nonalcoholic Fatty Liver Disease in High-Fat Diet-Induced Mice
Source: Nutrients. 2023 Apr 21;15(8):2005. doi: 10.3390/nu15082005 (PMC10143775; doi:10.3390/nu15082005)

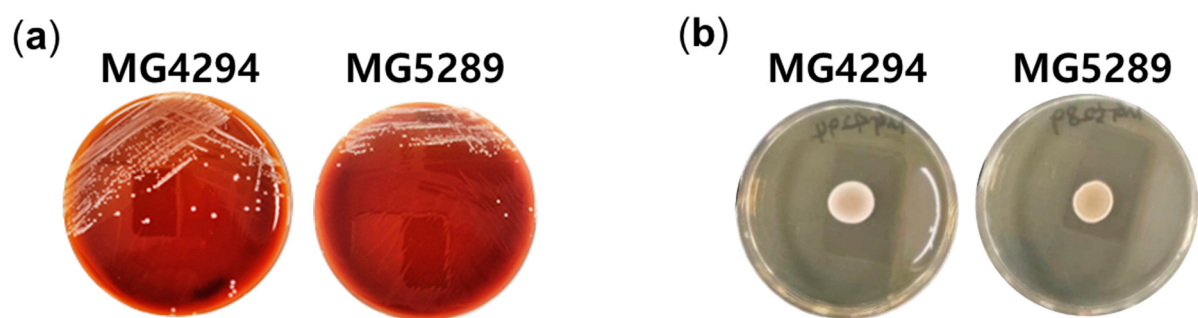

**Figure S1.** Hemolysis (a) and BSH activities (b) of *L. fermentum* MG4294 and *L. plantarum* MG5289.

Supplement: Supplementary file 1 [file nutrients-15-02005-s001.zip › nutrients-2355586-supplementary.pdf]
